# Supplementary material for: Random and combinatorial mutagenesis for improved total production of secretory target protein in Escherichia coli
Source: Sci Rep. 2021 Mar 5;11:5290. doi: 10.1038/s41598-021-84859-6 (PMC7935960; doi:10.1038/s41598-021-84859-6)
Supplement: Supplementary file 1 — Supplementary Information. [file 41598_2021_84859_MOESM1_ESM.pdf]

Random and combinatorial mutagenesis for improved total production of secretory target protein in *Escherichia coli*

Supplementary material

David Gonzalez-Perez[1,2], James Ratcliffe[1], Shu Khan Tan[1], Mary Chen May Wong[1], Yi Pei Yee[1], Natsai Nyabadza[1], Jian-He Xu[3], Tuck Seng Wong[1,4*], Kang Lan Tee[1*]

[1]Department of Chemical & Biological Engineering, The University of Sheffield, Sir Robert Hadfield Building, Mappin Street, Sheffield S1 3JD, UK

[2]Moffitt Cancer Center & Research Institute, Department of Drug Discovery, Stabile Research Building,12902 Magnolia Dr, Tampa, FL 33612, USA

[3]Laboratory of Biocatalysis and Bioprocessing, State Key Laboratory of Bioreactor Engineering, East China University of Science and Technology, 130 Meilong Road, Shanghai 200237, People's Republic of China

[4]National Center for Genetic Engineering and Biotechnology, 113 Thailand Science Park, Phahonyothin Road, Khlong Luang, Pathum Thani 12120, Thailand

*Address correspondence to:

Dr. Kang Lan Tee

Email: k.tee@sheffield.ac.uk

Tel: +44 (0)114 222 7507

Or

Dr. Tuck Seng Wong

Email: t.wong@sheffield.ac.uk

Tel: +44 (0)114 222 7591

Table S1: Primers used in this study

Primer	Sequence (5’ → 3’)
NdeI-Tfu0937-F	GGAATTCCATATGACCAGCCAAAGCACCAC
EcoRI-Tfu0937-R	CCGGAATTCTCATTACTCTTGACCAAAAATACC
BamHI-Lipase6B-F	GATCGGATCCATGGCGGAGCACAACC
Lipase6B-EcoRI-R	GATCGAATTCTTAGTTCGTGTTCTGG
BamHI-RFP-F	ACGGGATCCGCGAGTAGCGAAGACG
EcoRI-RFP-R	ACCGGAATTCTTAAGCACCGGTGGAGTG
OsmY-DirEv-F	GTACCATATGACCATGACCCGTC
OsmY-DirEv-R	CCGCGGATCCGCTACCTTTGGTCTTC
OsmY-V191E-FP	GTTGACGGCGAGAAGAGCGTTAAAAACGATCTGAAGA CCAAAGGTA
OsmY-V191E-RP	AACGCTCTTCTCGCCGTCAACCGCTTTCGCGATGCTCT CCGCACGA
OsmY-S154R-FP	ATCGTTCCGCGCCGTCACGTGAAGGTTGAAACCACCG ACGGCGTGG
OsmY-S154R-RP	CACGTGACGGCGCGGAACGATATCGTCCGCCAGCAGT TTCGCCTTA
OsmY-L6P-FP	ATGACCCGTCCGAAGATTAGCAAAACCCTGCTGGCGG TGATGCTGA
OsmY-L6P-RP	GCTAATCTTCGGACGGGTCATGGTCATATGTATATCTC CTTCTTAA

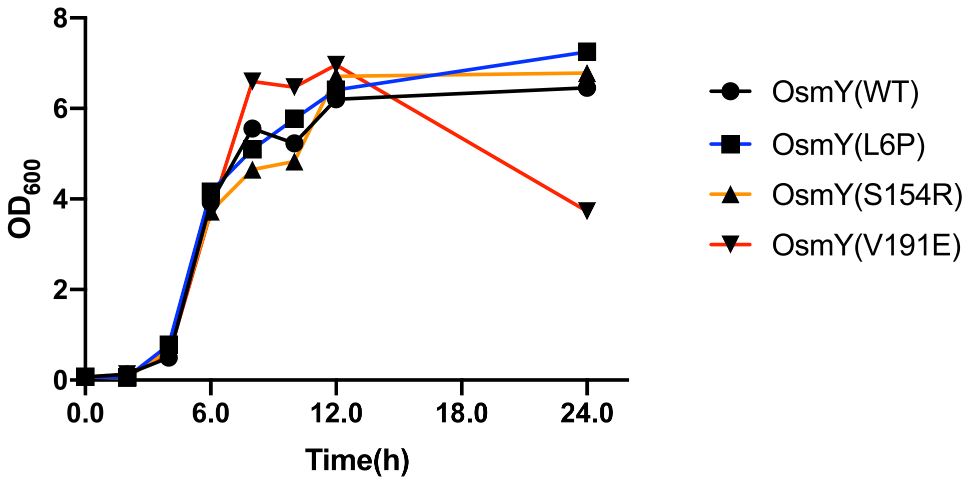

Figure S1: Growth curve of single site-directed mutants measured at 600 nm. Cells were cultivated in 20-mL flask cultures using 2×TY auto-induction media at 37 °C. Culture was inoculated using 1:100 dilution and 0.5 mL culture was sampled at various time points for cell density (OD_{600}) measurement. Wildtype and variants are represented using different colours; OsmY(WT) – black line, OsmY(L6P) – blue line, OsmY(S154R) – orange line and OsmY(V191E) – red line.

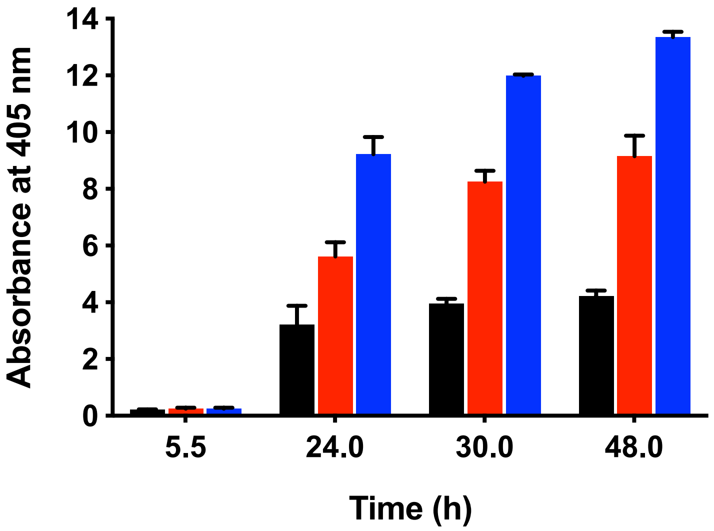

Figure S2: Characterization of improved variants in 50-mL flask cultures. The activity of extracellular OsmY-Tfu0937 was determined with the pNPG assay using an absorbance at 405 nm. Cultures were inoculated using 1:100 dilution. OsmY(WT) type and variants were expressed using C41(DE3) in 2TY auto-induction media and at 37 °C and 200 rpm. The activities of wildtype and variants are represented using different colours; OsmY(WT) (black), OsmY(TOA4) (red) and OsmY(M3) (blue). Experiments were performed in triplicates.

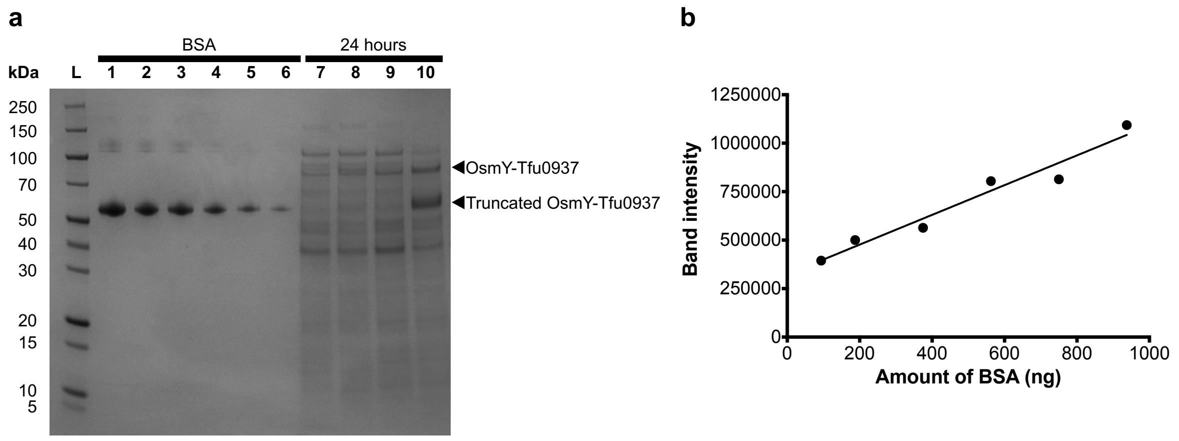

Figure S3: **(a)** SDS-PAGE of BSA standards and spent medium from 24 h cultivation. Lane L: protein marker, Lane 1: 250 µg/mL BSA, Lane 2: 200 µg/mL BSA, Lane 3: 150 µg/mL BSA, Lane 4: 100 µg/mL BSA, Lane 5: 50 µg/mL BSA, Lane 6: 25 µg/mL BSA, Lane7: OsmY(WT)-Tfu0937 in BL21(DE3), Lane 8: OsmY(M3)-Tfu0937 in BL21(DE3), Lane 9: OsmY(WT)-Tfu0937 in C41(DE3), Lane 10: OsmY(M3)-Tfu0937 in C41(DE3). **(b)** Calibration curve for protein quantification, obtained by densitometry of the BSA standards in (a) using ImageJ software.

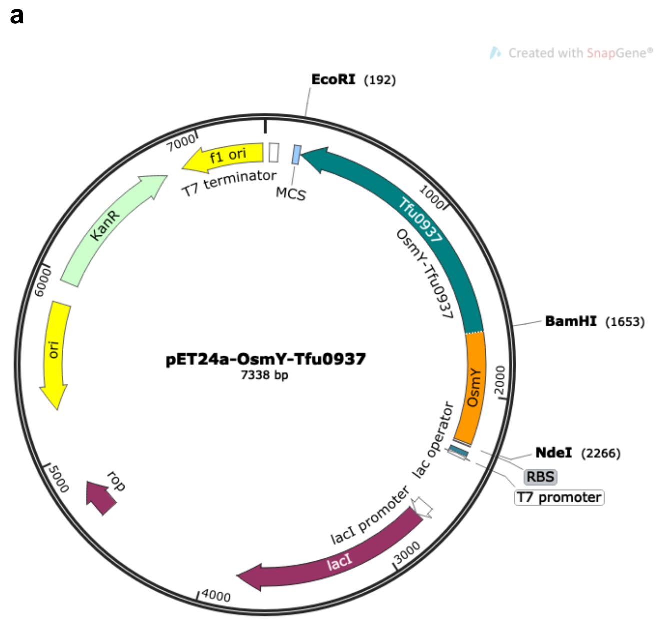

b

```
MTMTRLKISKTLLAVMLTSAVATGSAYAENNAQTT
NESAGQKVDSSMNKVGNFMDDSAITAKVKAALVDH
DNIKSTDISVKTDQKVVTLSGFVESQAQAEEAVKV
AKGVEGVTSVSDKLHVRDAKEGSVKGYAGDTATTS
EIKAKLLADDIVPSRHVKVETTDGVVQLSGTVDSQ
AQSDRAESIAKAVDGVKSVKNDLKTKGSGSTSQST
TPLGNLEETPKPDIRFPSDFVWGVATASFQIEGST
TADGRGPSIWDTFCATPGKVENGDTGDPACDHYNR
YRDDVALMRELGVGAYRFSIAWPRIQPEGKGTPVE
AGLDFYDRLVDCLLEAGIEPWPTLYHWDLPQALED
AGGWPNRDTAKRFADYAEIVYRRLGDRITNWNTLN
EPWCSAFLGYASGVHAPGRQEPAAALAAAHHLMLG
HGLAAAVMRDLAGQAGRSVRIGVAHNQTTVRPYTD
SEADRDAARRIDALRNRIFTEPLVKGRYPEDLIED
VAAVTDYSFVQDGDLKTISANLDMMGVNFYNPSWV
SGNRENGGSDRLPDEGYSPSVGSEHVVEVDPGLPV
TAMGWPIDPTGLYDTLTRLANDYPGLPLYITENGA
AFEDKVVDGAVHDTERIAYLDSHLRAAHAAIEAGV
PLKGYFAWSFMDNFEWALGYGKRFGIVHVDYESQT
RTVKDSGWWYSRVMRNGGIFGQE**
```

Figure S4: **(a)** Plasmid map of pET24a-OsmY-Tfu0937. **(b)** Amino acid sequence of OsmY-Tfu0937. The OsmY wildtype sequence is shown in orange, Tfu0937 sequence in green, and GSGS linker sequence in black.
